# Synergistic epistasis enhances the co-operativity of mutualistic interspecies interactions

**DOI:** 10.1038/s41396-021-00919-9

**Published:** 2021-02-21

**Authors:** Serdar Turkarslan, Nejc Stopnisek, Anne W. Thompson, Christina E. Arens, Jacob J. Valenzuela, James Wilson, Kristopher A. Hunt, Jessica Hardwicke, Adrián López García de Lomana, Sujung Lim, Yee Mey Seah, Ying Fu, Liyou Wu, Jizhong Zhou, Kristina L. Hillesland, David A. Stahl, Nitin S. Baliga

**Affiliations:** 1 Institute for Systems Biology, Seattle, WA 98109 USA; 2 Civil and Environmental Engineering, University of Washington, Seattle, WA 98195 USA; 3 Department of Biology, Portland State University, Portland, OR 97201 USA; 4 University of Iceland, Reykjavík, Iceland; 5 Division of Geological and Planetary Sciences, California Institute of Technology, Pasadena, CA 91125 USA; 6 Biological Sciences, University of Washington Bothell, Bothell, WA 98011 USA; 7 Institute for Environmental Genomics and Department of Microbiology & Plant Biology, University of Oklahoma, Norman, OK 73072 USA

**Keywords:** Microbial ecology, Population genetics, Symbiosis, Population dynamics, Molecular evolution

## Abstract

Early evolution of mutualism is characterized by big and predictable adaptive changes, including the specialization of interacting partners, such as through deleterious mutations in genes not required for metabolic cross-feeding. We sought to investigate whether these early mutations improve cooperativity by manifesting in synergistic epistasis between genomes of the mutually interacting species. Specifically, we have characterized evolutionary trajectories of syntrophic interactions of *Desulfovibrio vulgaris* (*Dv*) with *Methanococcus maripaludis* (*Mm*) by longitudinally monitoring mutations accumulated over 1000 generations of nine independently evolved communities with analysis of the genotypic structure of one community down to the single-cell level. We discovered extensive parallelism across communities despite considerable variance in their evolutionary trajectories and the perseverance within many evolution lines of a rare lineage of *Dv* that retained sulfate-respiration (SR+) capability, which is not required for metabolic cross-feeding. An in-depth investigation revealed that synergistic epistasis across pairings of *Dv* and *Mm* genotypes had enhanced cooperativity within SR− and SR+ assemblages, enabling their coexistence within the same community. Thus, our findings demonstrate that cooperativity of a mutualism can improve through synergistic epistasis between genomes of the interacting species, enabling the coexistence of mutualistic assemblages of generalists and their specialized variants.

## Introduction

Syntrophic interactions between bacteria and archaea are a major driver of anaerobic transformations of >1 gigaton/year of C into methane, which is ~30 times more potent than CO_2_ as a greenhouse gas [1]. Across diverse anoxic environments, including anaerobic reactors, animal guts, ocean and lake sediments and soils, in the absence of respirable electron acceptors, such as nitrate and sulfate, diverse syntrophs partner with methanogens to oxidize organic material. Syntrophy can be either obligate or facultative, for example, although oxidation via sulfate respiration (SR) is energetically favorable compared to syntrophy, many sulfate-reducing bacteria are facultative syntrophs that conditionally engage in syntrophy with methanogens in the absence of sulfate [2].

Conditional switching between syntrophy and SR is energetically expensive, requiring the differential regulation of thousands of genes [3]. Not surprisingly, frequent fluctuations between SR and syntrophy was demonstrated to be energetically unsustainable for a coculture of *Desulfovibrio vulgaris* Hildenborough (*Dv*) and *Methanococcus maripaludis* S2 (*Mm*) [3]. By contrast, prolonged laboratory evolution of the same community under obligate syntrophy conditions resulted in significantly improved growth and stability within 300 generations, but at the expense of loss of independence through the erosion of SR [4, 5]. Another striking discovery was that a subpopulation of cells capable of respiring sulfate (SR+) persisted in low frequency within the dominant non-sulfate respiring (SR−) populations for most evolved lines [5]. Persistence of SR+ cells during syntrophy suggested that they may be adapted to a narrow niche that the dominant SR− population is unable to exploit effectively. One hypothesis is that SR+ and SR− have specialized growth dynamics, allowing for their coexistence, e.g., as *r*- and *K*-strategists in a seasonal environment [6–8]. Another consideration is that the Black Queen Hypothesis (BQH) can explain the persistence of the SR+ population [9]. In this hypothesis, the SR+ population subsists by producing a costly metabolite that SR− cells cannot. Dependency of SR− cells on the metabolite prevents them from completely excluding SR+ cells even though the SR+ cells pay the cost for the metabolite. Given that mutations in many pathways in the two organisms could have improved the mutualism, this also raised the possibility that distinct interactions between SR− and SR+ populations and different subpopulations of evolved *Mm* (partner choice [10]) could have independently increased the productivity of syntrophy in each of the two subpopulations. Notably, naturally occurring polymorphisms in ion-translocating CooK subunit of the membrane-bound COO hydrogenase of *Dv* are known to be essential for mutualism with *Mm*, demonstrating that partner choice is important in promoting facultative syntrophic interactions [11].

**Fig. 1:**
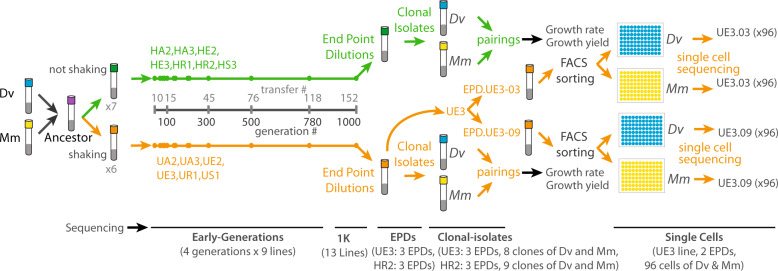
Overview of directed laboratory evolution to probe evolutionary signatures for syntrophic cocultures of *Dv* and *Mm*. Thirteen independent cocultures were subjected to laboratory evolution with and without shaking as described before [5]. DNA samples were collected across generations, end-point-dilutions (EPDs), clonal isolates, and single cells to identify genomic alterations. In addition, clonal isolates were paired in varied combinations in order to determine growth rate and yield for cocultures. Number of samples sequenced are indicated at the bottom.

In coevolved microbial interactions, the fitness of individual organisms may depend on the genotypes of both partners [12]. These epistatic interactions between two genomes of different species can be described as a form of “intergenomic interaction” [13]. Intergenomic interactions can manifest in overall fitness of the community that is greater (synergistic) or less (antagonistic) than the sum of fitness contributions of each interacting organism. Prior work using synthetic communities of *yeast*, *E. coli* and *Salmonella enterica* have demonstrated the potential for synergistic epistasis to improve interspecies cooperation [14–16]. Here, we have investigated whether intergenomic synergistic epistasis can indeed emerge during evolution to enhance cooperativity (combined metabolic activity of interacting microorganisms for efficient cross-feeding) and productivity of a mutualism that plays a central role in biogeochemical C cycling across diverse environments. Further, we have also investigated whether intergenomic epistasis might also contribute to the coexistence of assemblages of a generalist and their specialized variants within the same evolution line. Briefly, we tracked the longitudinal patterns in which mutations accumulated in *Dv* and *Mm* across 1000 generations of nine independent evolution lines. From the 1 K generation of two lines, we generated simplified communities through serial end-point dilutions (EPDs). Bulk sequencing of the simplified communities revealed how parental mutations were segregated into each EPD, and discovered evidence for the existence of interactions among specific evolved lineages of *Dv* and *Mm*, within the same evolution line. Through single-cell sequencing, we then inferred and characterized interactions within a SR+ and a SR− EPD derived from the same parental population. Finally, we quantified growth rate, yield, and cooperativity of each EPD, and pairings of evolved and ancestral clonal isolates of *Dv* and *Mm*. These analyses uncovered synergistic epistasis as a plausible mechanism for the increased cooperativity of mutualistic interactions within EPDs, giving likely mechanistic explanation for coexistence of SR+ and SR− assemblages in the same community (Fig. 1).

## Results

### Distribution, frequency, and functional implications of mutations during laboratory evolution of obligate syntrophy

We evaluated whether the selection of mutations in the same genes (i.e., “parallel evolution” [17]) had contributed to improvements in syntrophic growth of *Dv* and *Mm* across independent evolution lines, all of which started with the same ancestral clone of each organism. The goal of this analysis was to focus on generalized strategies for adaptation to syntrophy, irrespective of the culturing condition so we investigated parallelism across both U and H lines. Based on the number of mutations (normalized to gene length and genome size) in *Dv* and *Mm* across 13 evolved lines (six lines designated U for “uniform” conditions with continuous shaking and seven H lines for “heterogenous” conditions without shaking), we calculated a G-score [18] (“goodness-of-fit”, see “Methods” section [18]) to assess if the observed parallel evolution rate was higher than expected by chance. The “observed G-score” was calculated as the sum of G-scores for all genes in the genome of each organism; mean and standard deviation of “expected G-scores” were calculated through 1000 simulations of randomizing locations of observed numbers of mutations across the genome of each organism. The observed total G-score for *Dv* (1092.617) and *Mm* (805.02) was significantly larger than the expected mean G-score (*Dv*: 798.19 ± 14.99, *Z* = 19.63 and *Mm*: 564.83 ± 15.95, *Z* = 15.06), demonstrating significant parallel evolution across lines.

**Fig. 2:**
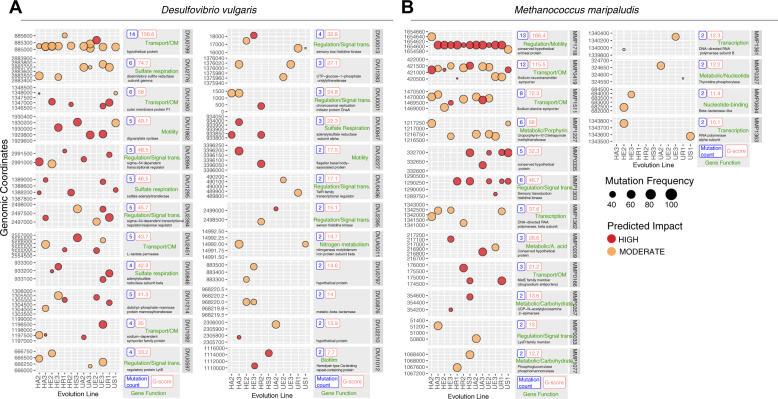
Frequency and location of high G-score mutations in *Dv* (A) and *Mm* (B) across 13 independent evolution lines. SnpEff predicted impact of mutations* are indicated as moderate (orange circles) or high (red circles) with the frequency of mutations indicated by node size. Expected number of mutations for each gene was calculated based on the gene length and the total number of mutations in a given evolution line. Genes with parallel changes were ranked by calculating a G (goodness of fit) score between observed and expected values and indicated inside each panel. Mutations for each gene are plotted along their genomic coordinates (vertical axes) across 13 evolution lines (horizontal axes). Total number of mutations for a given gene is shown as horizontal bar plots. [*HIGH impact mutations: gain or loss of start and stop codons and frameshift mutations; MODERATE impact mutations: codon deletion, nonsynonymous in coding sequence, change or insertion of codon; low impact mutations: synonymous coding and nonsynonymous start codon].

With the exception of five high G-score genes (DVU0597, DVU1862, DVU0436, DVU0013, and DVU2394), which were mutated during long term salt adaptation of *Dv* [19], mutations in other high G-score genes appeared to be putatively specific to syntrophic interactions. Altogether, 24 genes in *Dv* and 16 genes in *Mm* associated with core processes had accumulated function modulating mutations across at least 2 or more evolution lines (Fig. 2 and Supplementary Table S1). Signal transduction and regulatory gene mutations (seven in *Dv* and six in *Mm*) represented 19.9% and 27.2% of all mutations in *Dv* and *Mm*, respectively, similar to long term laboratory evolution of *E. coli* [18], potentially because their influence on the functions of many genes [20, 21]. We also observed missense and nonsense mutations in outer membrane and transport functions (four genes in *Dv* and three genes in *Mm*). For example, the highest G-score gene in *Dv*, DVU0799—an abundant outer membrane porin for the uptake of sulfate and other solutes in low-sulfate conditions [22], was mutated early across all lines, with at least two missense mutations in UE3 (S223Y) and UA3 (T242P). Notably, the regulator of the archaellum operon (MMP1718) had the highest G-score with frameshift (11 lines) and nonsynonymous coding (2 lines) mutations [23]. Similarly, two motility-associated genes of *Dv* (DVU1862 and DVU3227) also accumulated frameshift, nonsense and nonsynonymous mutations across 4 H and 3 U lines. Together, these observations were consistent with other laboratory evolution experiments performed in liquid media [24], suggesting that retaining motility has a fitness cost during syntrophy [25, 26].

Consistent with our previous observation that obligate mutual interdependence drove the erosion of metabolic independence of *Dv* [5, 27], mutations in SR genes were among the top contributors to the total G-score in *Dv* (DVU2776 (74.7), DVU1295 (46.5), DVU0846 (42.9), and DVU0847 (22.3)). However, it was intriguing that DVU2776 (DsrC), which catalyzes the conversion of sulfite to sulfide, the final step in SR, accumulated function modulating but not loss-of-function mutations across six lines. The functional impact of these mutations is not clear but it is possible that these changes might alter previously suggested alternative roles for this gene, including electron confurcation for the oxidation of lactate [28], sulfite reduction, 2-thiouridine biosynthesis and possibly gene regulation [29].

### Analysis of temporal appearance and combinations of mutations across evolution lines

**Fig. 3:**
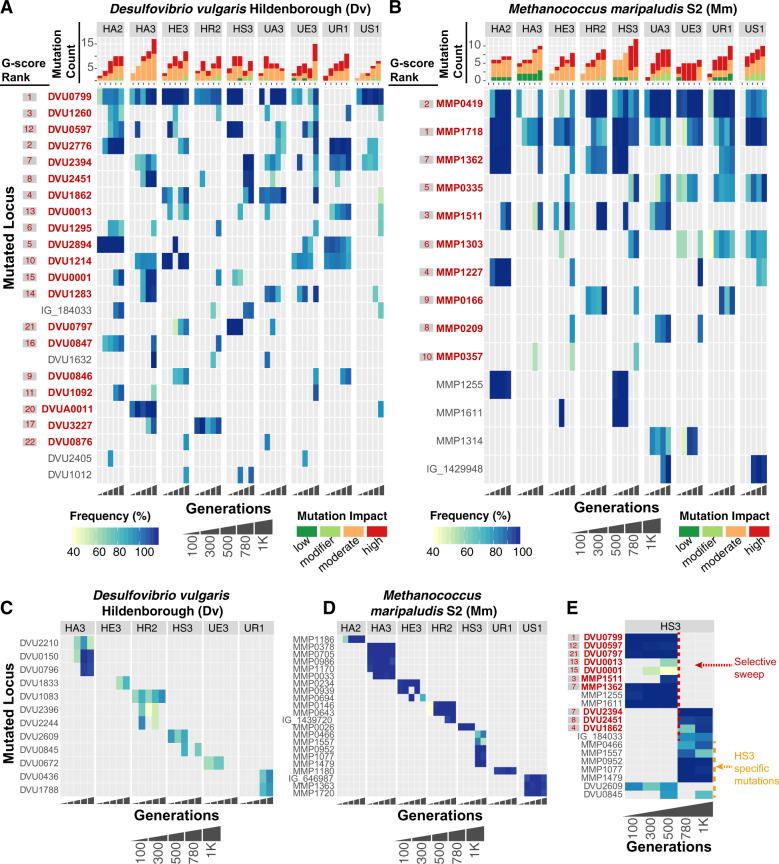
Frequency and time of appearance of mutations through 1 K generations of laboratory evolution lines of *Dv* and *Mm* cocultures. The heat maps display frequency of mutations in genes (rows) in *Dv* (**A**) and *Mm* (**B**) in each evolution line, ordered from early to later generations (horizontal axis). High G-score genes are shown in red font and their G-score rank is shown to the left in gray shaded box, also in red font. Bar plots above heat maps indicate total number of mutations in each generation and the color indicates impact of mutation. Use “Frequency”, “Generations”, and “Mutation impact” key below the heat maps for interpretation. Mutations that were unique to each evolution line is shown in (**C**, **D**) for *Dv* and *Mm*, respectively. **E** The heatmap illustrates a selective sweep across both organisms in line HS3.

Growth characteristics of all evolution lines improved by the 300th generation [4], and in some lines even before the appearance of SR− mutations, indicating that mutations in other genes had also contributed to improvements in syntrophy. Each evolution line had at least 8 and up to 13 out of 24 high G-score mutations in *Dv*, while *Mm* had mutations in at least 5 and up to 10 out of 16 high G-score genes. We interrogated the temporal order in which high G-score mutations were selected and the combinations in which they co-existed in each evolution line to uncover evidence for epistatic interactions in improving obligate syntrophy. Indeed, missense mutations in DsrC (DVU2776) were fixed simultaneously with the appearance of loss of function mutations in one of two sigma 54 type regulators (DVU2894, DVU2394) in lines HA2, and UR1 (*P* = 5.40 × 10^−5^). In rare instances, we also observed that some high G-score mutations co-occurred across evolution lines, e.g., two U- and one H-line consistently showed for at least two time points a mutation in DVU1283 (GalU) coexisting with mutations in DVU2394 (*P* = 5.04 × 10^−3^). More commonly, the combinations of high G-score gene mutations varied across multiple lines. In fact, no two lines possessed identical combination of high G-score gene mutations (Fig. 3A, B), and many high-frequency mutations were uniquely present or absent in different lines (Fig. 3C, D).

Mutations in high G-score genes appeared consistently in all evolution lines (*P* < 1.82 × 10^−2^), although in a different temporal order in each line. Mutations in the same high G-score genes appeared at different times (e.g., whereas mutations in SR gene DVU0847 first appeared in the 300th generation of HA2, they appeared much later in HR2 and HA3) (Fig. 3A). Similar temporal patterns of appearance and co-occurrence of mutations were observed in *Mm* (Fig. 3B). We also discovered evidence for temporally nested fixations, wherein prior to fixation of a mutation from an earlier generation, another mutation selected in a later generation gradually increased in frequency towards fixation e.g., DVU0799, DVU0001, and DVU1283 in HA3 (*P* = 1.19 × 10^−3^); and MMP1718 and MMP0335 in UA3 (*P* = 1.81 × 10^−4^). Moreover, there were many cases of simultaneous fixation of mutations in multiple genes (e.g., DVU0799 and DVU1214 in HE3; MMP0378, MMP0705, MMP0986, and MMP1170 in HA2) suggesting that hitchhiking may be common [30, 31]. However, given that samples were only sequenced every 250 generations, we cannot rule out the possibility of each mutation sweeping separately during that time interval. These observations lead us to conclude that mutations that were commonly selected may simply have additive effects on fitness, arising at different times in different populations because of chance (i.e., they became available for selection at different times in different populations).

The longitudinal analysis revealed a cross-species selection event that resulted in the replacement of the dominant clones of both species with new clones containing different mutations. Between generations 500 and 780 of HS3, the dominant *Dv* (harboring high G-score mutations DVU0799, DVU0597, DVU0797) and *Mm* (harboring dominant mutations in MMP1255, MMP1611, MMP1362, and MMP1511) clones disappeared (Fig. 3E). At the same time a new *Dv* clone with mutations in DVU2394, DVU2451, DVU1862, and intergenic region IG_184033, and a new *Mm* clone with mutations in MMP0952, MMP1077, and MMP1479 were selected (Fig. 3E). One explanation for this phenomenon is that, coincidentally, rare clones in both *Dv* and *Mm* acquired beneficial mutations and outcompeted dominant clones in the same 250 generation interval of evolution. Another possibility is that selection of a new dominant clone in one species changed the selection environment for the other, allowing its rare clone to take over. Which species might have started this process is unclear because there are no samples available in the 250 generations during which the sweep occurred. However, information about the new mutations, their functions, and parallel evolution may provide hypotheses. The novel mutations in DVU2394 (a sigma 54-dependent transcriptional regulator) and DVU2451 (a lactate permease) co-occurred in at least two lines including HS3 (*P* = 3.93 × 10^−2^) and appeared individually in only three other lines, suggesting that the two genes might be beneficial and also functionally coupled in the context of promoting syntrophy. Notably, we demonstrate later through single-cell analysis that mutations in DVU2394 occurred subsequent to mutations in DVU2451, but exclusively in the SR− lineage within UE3. Interestingly SR− mutations were never selected through 1000 generations in HS3 line. Conversely, while mutations in DVU2394, DVU2451, and DVU1862, all co-occurred in UE3, they did not sweep through the population, underscoring how improvements to syntrophic interactions occurred through multiple distinct trajectories in terms of the order and combinations of mutation selection. In other words, this cross-species selective sweep occurred only in HS3, suggesting one of several features unique to this line was responsible, including simultaneous selection of mutations in DVU2394, DVU2451, and DVU1862, the overall mutational landscape of HS3 between generations 500 and 780, or mutations unique to HS3. Interestingly, fixed mutations that were observed only in HS3 were in *Mm* (MMP0952, MMP1077, MMP1479) and their appearance coincided with the selective sweep between 500 and 780 generations. Regardless of the mechanism, it is interesting that a new mutation(s) in *Mm* appears to have selectively swept high G-score mutations across both interacting organisms, strongly suggesting that the new *Mm* genotype conferred a fitness advantage to a specific lineage of genotypes in *Dv* that were in low abundance prior to the sweep.

### Characterization of evolutionary lineages and interspecies interactions in minimal assemblages at single-cell resolution

**Fig. 4:**
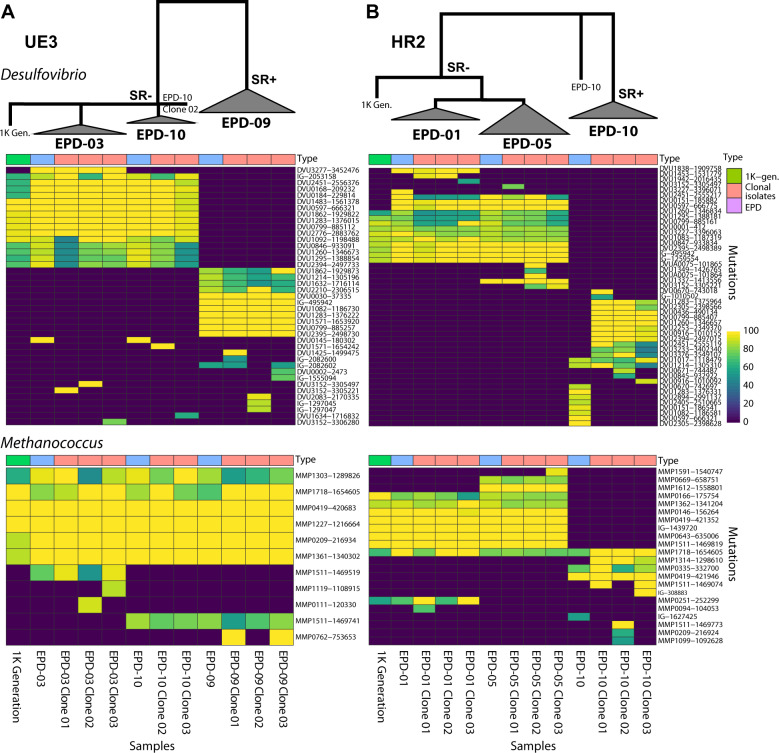
Genotype mapping of 1 K generation, EPDs and clonal isolates for two evolution lines. Heatmap displays frequency for each mutation (rows) in UE3 (**A**) and HR2 (**B**) across 1 K generation, EPDs, and clonal isolates (columns). The upper panel shows genotype map of *Dv* and the lower panel for *Mm*. The hierarchical tree indicates a simplified lineage map of mutations in *Dv* within each evolution line, with SR phenotypes indicated.

We performed EPDs from the 1 K generation of HR2 and UE3 lines to generate simplified sub-communities that represented minimal sets of genotypes with growth phenotypes comparable to the 1 K culture (see “Methods” section). While two EPDs from each line represented the dominant SR− subpopulation of the 1 K evolved line, we also recovered an SR+ subpopulation that co-existed within each line albeit at much lower abundance and below the detection limit of bulk mutation analysis of the parental culture (Fig. 4). Finally, we isolated evolved clones of each organism by streaking EPDs on agar plates containing nalidixic acid and neomycin, taking advantage of chromosomally integrated selection markers in *Dv* and *Mm*, respectively. Altogether, three clones of *Dv* and *Mm* from each EPD were isolated and re-sequenced. The distribution of unique mutations across EPDs and clonal isolates added evidence for coexistence of distinct lineages of one or both organisms within each evolved line. Logically, all high-frequency mutations in an asexual population must be linked on the same genetic background. As expected, all 15 high-frequency mutations detected in the 1 K generation of UE3 were present only in sub-communities with the SR− mutations (EPD-03 and EPD-10). By contrast, at least 11 mutated loci (ten genic and one intergenic) in the SR+ sub-community (EPD-09) were not detected in the SR− sub-communities or in 1 K bulk sequencing of UE3, demonstrating that the EPD-09 assemblage was made up of rare *Dv* lineages (Fig. 4A and Supplementary Table S2). Strikingly, both *Dv* and *Mm* lineages in the SR+ assemblage of HR2 were distinct from lineages in the SR− EPDs, and below detection limit in 1 K bulk sequencing (Fig. 4B, Supplementary Fig 1 and Supplementary Table S2). Thus, the existence of genotypically distinct subpopulations of *Dv* and *Mm* with the parental growth phenotype suggested that specific interactions across multiple evolved genotypes of the two organisms could have emerged during their syntrophic evolution.

We further investigated the evidence for specific interactions among evolved genotypes using single-cell sequencing of SR− (EPD-03) and SR+ (EPD-09) assemblages from UE3. We sorted, amplified, and re-sequenced the genomes of single cells of *Dv* (94 from EPD-03, and 94 from EPD-09) and *Mm* (87 from EPD-03, and 72 from EPD-09) to reconstruct lineages of both organisms within each EPD ([32] and “Methods” section, Supplementary Table S3). Using stringent cutoffs (fold coverage ≥ 8, number of cells with mutation ≥2, frequency ≥ 80%) and consensus mutation calling using varscan [33], GATK [34], and Samtools [35]_,_ we identified across single cells of *Dv* 16 of 17 and 3 of 12 mutations detected in bulk sequencing of EPD-03 and EPD-09, respectively. Similarly, we identified across *Mm* single cells seven of seven and six of seven mutations from bulk EPD-03 and EPD-09, respectively.

**Fig. 5:**
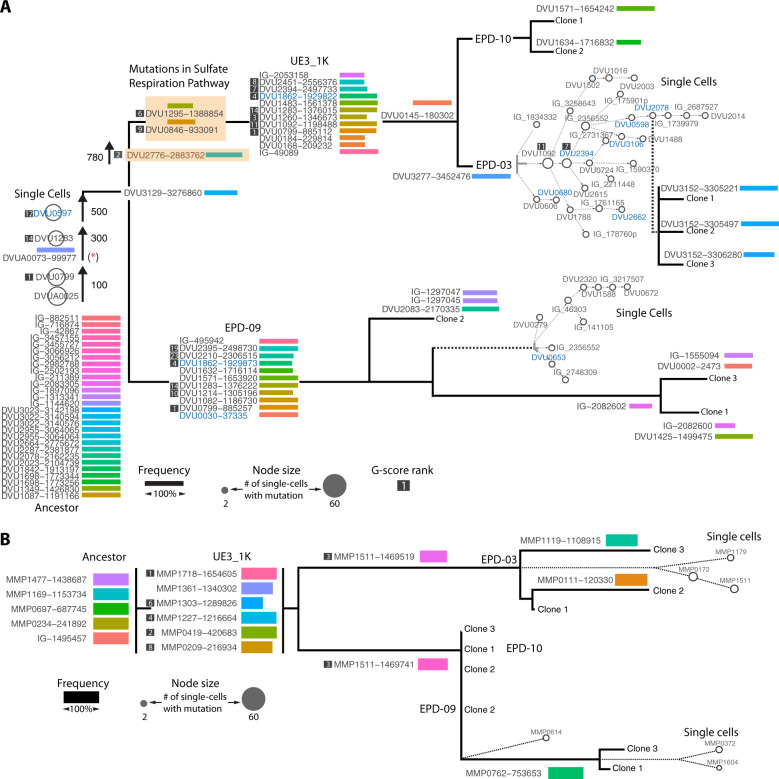
Lineage map of mutational events deciphered through sequencing of up to 96 single cells of (A) *Dv* and (B) *Mm* from EPD-03 and EPD-09, cross-referenced with longitudinal bulk sequencing of UE3, EPDs and sequencing of clonal isolates. Temporal ordering of mutations in the trunk is based on their order of appearance in longitudinal sequencing data across generations. Unique mutations within each lineage are shown together with frequency (length of bars). The single-cell lineage tree for each EPD was constructed using the algorithm SCITE and shown in the context of the parent EPD and linked to clonal isolates. (See Supplementary Figs. 2 and 3 for details). Mutation names for regulatory or signal transduction genes are colored in blue and SR-related genes are indicated with an orange shaded box. An asterisk indicates mutation in a plasmid gene that was not detected in single cells potentially due to loss of plasmid. Of the total 11 high G-score *Dv* genes in the 1 K generation of UE3, just three were observed in both EPDs. Note, the three high G-score genes DVU1862, DVU2394, and DVU0799 had mutations in different locations in the two EPDs. High G-score genes that were only observed in EPD-03 were DVU2451, DVU1260, and DVU1092, and those unique to EPD-09 were DVU2395, DVU2210, and DVU1214. In addition, SR− mutations in DVU0846 and DVU1295 were unique to EPD-03, appearing after 780 generations, and were present across single cells and all clonal isolates.

Using a mutation lineage inference algorithm SCITE [36] and cross-referencing with longitudinal sequencing data from 100, 300, 500, 780, and 1000 generations, bulk sequencing of EPDs, single-cell sequencing, and sequencing of clonal isolates, we reconstructed the lineage and timeline of mutations that shaped the evolution of syntrophy in SR− and SR+ communities within UE3 (see “Methods” section) (Fig. 5 and Supplementary Figs. 2–3). As expected, the two EPDs shared a core lineage of events that included sequential accumulation of high G-score mutations in the early stages of evolution in both organisms. While the *Mm* lineages across EPDs had few differences, lineages of *Dv* were strikingly different across the SR− and SR+ communities. The SR− mutations in the EPD-03 lineage were followed by selection of mutations in at least six regulators, and complex radiating branches with many coexisting sub-clones, suggesting that loss of SR in the EPD-03 line might have promoted the selection of mutations in regulatory genes. Altogether, the observation that dominant lineages were excluded in the minimal community assemblages of EPD-09, demonstrates the coexistence of distinct high abundance (SR−) and low abundance (SR+) lineages within the same evolved population (Figs. 4 and 5 and Supplementary Figs. 1–3). A surprising observation is that the SR+ clone that remained in the population subsequent to the evolution of SR− was not simply the dominant clone without the SR− mutation. Instead, it was a rare genotype with different mutations from the dominant population.

#### Investigation of cooperativity and synergistic interspecies interactions

**Fig. 6:**
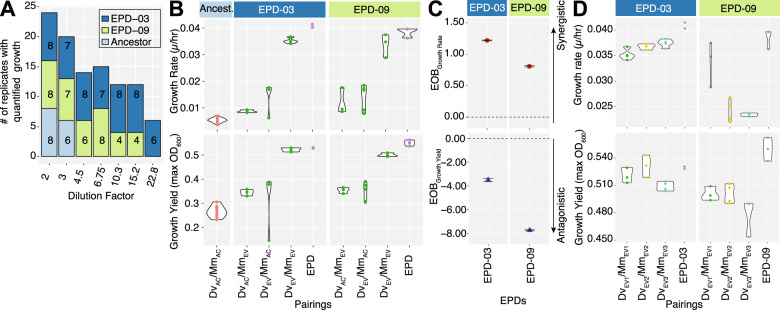
Growth rate, yield and cooperativity of EPDs, and clonal isolate pairings. **A** A stacked barplot showing the number of replicates exhibiting growth for each EPD and the ancestral cocultures across a dilution series. **B** Growth rate and carrying capacity of pairings of ancestral and evolved clonal isolates of *Dv* and *Mm* from EPD-03 and EPD-09. **C** Excess-Over-Bliss analysis for estimating synergistic and antagonistic interactions of *Dv*/*Mm* clonal isolate pairings. **D** Growth rate and yield for three evolved *Dv*/*Mm* pairings from each EPD.

We performed a density dilution assay to investigate the evidence for improved cooperativity due to interactions among specific genotypes of *Dv* and *Mm* within microbial community assemblages of the two EPDs [37, 38]. Briefly, we generated a dilution series of both EPD and ancestral cell lines in 96-well plates and determined growth rate, carrying capacity, and a minimal cell density that supported syntrophic population growth (See “Methods” section, Supplementary Fig. 4). Both EPDs could initiate growth at significantly lower cell density relative to the ancestral coculture. EPD-03 initiated growth at a 1.5-fold lower cell density with faster growth rate and lower carrying capacity relative to EPD-09, explaining how the two EPDs co-existed in vastly different proportions in UE3 (>80% EPD-03 vs, <1% EPD-09, Fig. 6A). These data make a compelling case for the emergence of increased cooperativity among *Dv* and *Mm* lineages during the evolution of syntrophy.

We investigated whether the increased cooperativity could have emerged through synergistic interactions between *Dv* and *Mm* lineages by characterizing individual and combined contributions of the two evolved partners towards improved growth characteristics. By comparing pairs of evolved and ancestral clones (*Dv*_EV_ × *Mm*_EV_, *Dv*_Ac_ × *Mm*_Ev_ and *Dv*_Ev_ × *Mm*_Ac_, *Dv*_Ac_ × *Mm*_Ac_), we determined that each evolved clonal isolate had contributed individually to significant improvement in growth rate and yield (Fig. 6B and Supplementary Table S4). The improvements were maximal, and comparable to growth characteristics of the parental EPD, when both partners in the interacting pair were evolved clonal isolates (*Dv*_Ev_ × *Mm*_Ev_). This result demonstrated unequivocally that increased cooperativity had emerged from synergistic interactions between the evolutionary changes in both species within each EPD, with proportional antagonistic effect on growth yield [39] (Fig. 6C). The higher growth rate of EPD-03 and higher carrying capacity of EPD-09 (both relative to the other EPD) gives mechanistic insight into coexistence of SR− and SR+ sub-communities as a *r*- and *K*-strategists, respectively (Fig. 6D, Supplementary Fig. 4). Notably, the few mutations that differentiate genotypes of each clonal isolate appear to manifest in variation in growth rate and yield, demonstrating that productivity of *Dv*_*EV*_ × *Mm*_*EV*_ interactions are genotype-specific, even within the same EPD (Fig. 6D, Supplementary Fig. 5, Supplementary Table S2).

## Discussion

We sought to understand the evolutionary trajectories that increase the productivity of interspecies interactions of *Dv* with *Mm* in an obligate syntrophic association, while retaining a small subpopulation that can respire sulfate. To do so, we combined a broad survey of all the mutations accumulated over the first 1000 generations of nine independently evolved communities with an in-depth study of the genotypic structure of one community down to the single-cell level. These data showed a high level of parallelism across communities despite considerable variance across populations in their evolutionary trajectories. A detailed view of one community revealed the perseverance and evolution of a rare lineage that maintained its ability to respire sulfate while the rest of the population did not. Growth experiments with clones and subpopulations demonstrated that the SR+ and SR− *Dv* subpopulations both cooperate more efficiently with corresponding evolved *Mm* partners, allowing them to grow at lower starting densities than the ancestors. The collective action of clones within each subpopulation has a synergistic effect on population growth rate and an antagonistic effect on yield. Finally, the different growth dynamics of SR− and SR+ evolved communities is consistent with how the two communities might coexist in vastly different proportions, likely as *r*- and *K*-strategists.

The evolutionary trajectory of a microbial population depends on the order in which mutations occur (chance), and the relative effects of the pool of mutations on fitness (selection) [40]. If the effects of each beneficial mutation are constant, meaning they do not vary in the presence of other polymorphisms or species, then all populations would eventually acquire the same mutations, even if they occur and are, therefore, selected in a different order. However, the effect of an allele on fitness may depend on epistasis, where the effect of an allele changes depending on alleles at other loci in the same genome [41]. In this case, the order in which mutations occur in different populations could affect their overall trajectories. This relationship between fitness and the possible combinations of genetic variants is called an adaptive landscape, and has been the subject of intense research [42–45].

In the present work, we investigated evolutionary trajectories of not just one but two species that rely on one another for survival. One might expect the interaction to amplify the effects of chance if the adaptive landscape is affected by genetic changes in the partner population (coevolution; [46, 47]), and those partner genetic changes depend on chance. In an extreme case, this situation could send each population down completely different trajectories, with very little parallelism. However, that is not the result that was observed here. The discovery of high G-score mutations in both *Dv* and *Mm* made a compelling case that parallel evolution was a dominant driver of productive obligate syntrophy (Fig. 2). It is important to emphasize that in order to increase statistical power, our assessment of parallelism was based on the analysis of all mutations pooled from two different evolution environments (U: Uniform and H: Heterogenous). While the two treatments were different in terms of mixing, there were also many similarities, including the chemical composition of the media, the incubation temperature, and the fact that the species were forced to rely on syntrophy for survival instead of growing alone with different metabolism. In fact, observation of similar numbers of mutations in both U and H lines suggests that substantial adaptation happened in both environments. That being said, we also detected parallelism, albeit with lower statistical significance, when the analysis was repeated with either U or H lines, separately (Supplementary Fig. 1). Genes associated with parallel evolution are usually under strong selection [48], and implicated as a major driver of evolution of bacteria [49], phages [50] and microbial communities [51–54]. To our knowledge, this is the first demonstration of a role for parallel evolution in driving mutualism across metabolically coupled species.

While the high number of G-score mutations suggests that parallel changes conferred fitness benefits across a range of genotypes [50], in some populations these high G-score mutations were selected in a different order, suggesting epistasis did not substantially constrain the timing of selection of a given high G-score mutation. Many differences between populations in mutation order could have occurred due to the chance occurrence of mutations at different times in different populations [55]. However, we cannot rule out the possibility that epistasis and evolutionary history caused some of the differences between populations [56, 57]. For example, it is possible that a mutation unique to HS3 precluded or significantly delayed erosion of SR in this coculture.

Intergenomic epistasis is a pre-requisite for coevolution and the synergistic epistasis observed in this study could have caused or resulted from coevolution. There are a few reasons to believe that each species is not evolving independently in a constant environment consisting of another species, and that some evolutionary changes likely resulted from genetic interactions between specific evolved genotypes of *Dv* and *Mm*. The most striking example was observed in community HS3. In this community, it seems that one or more new mutations in one partner affected the fitness of the dominant clone of the other partner, causing it to decrease in frequency below the limits of detection, while a new clone arose. A plausible hypothesis for this intergenomic epistasis is that the selective sweep likely occurred due to the loss of function mutations in MMP1077, a putative phosphomannomutase, which re-directed monosaccharides towards synthesis of exopolysaccharides to promote intercellular interactions through clumping or flocculation [58]. Regardless of the mechanism, the interesting observation is that both *Dv* and *Mm* clones that were in low frequency swept through the entire population, indicating preferential interactions among those clones. This two-population selective sweep suggests epistasis between specific genotypes of the two interacting species. This hypothesis of partner choice was also supported by the observation that growth rates and yields differed between some pairings of clones within a population, demonstrating variation in effectiveness of cooperation.

While most adaptive mutations rose to fixation, SR mutations did not show complete penetrance. In fact, previously we reported that SR+ populations were readily obtained from every evolved coculture even after 1300 generations [5] and through characterization of EPDs and single cells we have re-confirmed the presence of rare SR+ subpopulations in most evolved populations. One explanation could be that SR+ cells persist because the maintenance of SR machinery allows them to produce a costly but essential metabolite. Leaking of this metabolite could allow SR− cells to survive without paying the cost of production, allowing them to flourish as long as SR+ cells and the leaked resource do not become scarce. In other words, these SR+ cells might act as “helpers” for the “beneficiary” SR− cells as stated by BQH [9]. High expression of SR genes even under syntrophic conditions [59] supports this hypothesis. However, a minimal assemblage that is entirely composed of SR− cells (EPD-03 of UE3 line) does better than assemblage of SR+ cells (EPD-09 of the same line) in cooperativity assays with no apparent growth defect indicating that SR+ cells do not play an essential role in supporting syntrophic growth of the population. In fact, the poor performance of EPD-09 relative to EPD-03 suggests that SR is too expensive to maintain and, therefore, undesirable during syntrophy. Moreover, individual SR− clonal isolates synergistically improved growth characteristics of cocultures upon pairing with evolved *Mm* further demonstrating that coexistence of SR+ and SR− populations cannot be explained solely by the BQH.

Alternatively, SR+ and SR− cells may be adapted to different niches that arise as a result of the seasonal changes in resources that recur in each transfer-cycle of the evolution experiment [8]. Specifically, growth dynamics of the two EPDs [higher growth rate (*r*) of EPD-03 and higher carrying capacity (*K*) of EPD-09] suggest that the faster-growing SR− lineages (*r*-strategists) can initiate growth at lower cell density and are, therefore, favored in early growth phase when resources are plentiful but fluctuating. The slower growing SR+ lineages (*K*-strategists) are favored in later stages of growth when the resources are limited but stable, and cell density is high [6]. Hence, these growth dynamics based on *r/K* tradeoffs might explain why SR+ populations are retained in the absence of sulfate. In the natural world, where sulfate availability varies over time, persistence of SR+ genotypes in the absence of sulfate may stabilize sulfate-reducing populations overall. In other words, it may be a bet-hedging strategy (similar to maintenance of subpopulations with COO hydrogenase polymorphisms [11, 60]) that might contribute to the success of *Dv* as a generalist that can conditionally switch between SR and syntrophy without the need for expensive gene regulatory changes [3].

It was significant that each of the two EPDs segregated a subset of high G-score mutations into simplified assemblages but retained growth rate and carrying capacity of the parental evolved population. This result demonstrated that multiple independent evolutionary strategies can coexist in the same population, albeit in vastly different proportions. Whether the distinct sets of *Dv* and *Mm* mutations within each EPD reflect coevolution will require additional experiments, including pairing with evolved populations from preceding generations [47]. Notwithstanding that caveat, the ability of evolved isolates of *Dv* and *Mm* to synergistically improve growth characteristics lends credibility to the claim that complementary genetic changes (e.g., in transport, regulation, and motility) enhanced metabolic coupling and cross-feeding between the two interacting organisms, significantly increasing their cooperativity.

The nature of cooperation in this syntrophic mutualism is unclear. On the surface, it seems like the fitness of *Dv* and *Mm* would be aligned and exploitation unlikely [61–63] because the production of hydrogen is a necessary byproduct of metabolism for *Dv* and the only energy source available for *Mm*. Efficient transfer of electrons through hydrogen is in the best interests of both species [64]. However, evolution could hypothetically change this situation by altering mechanisms of electron transfer, or through the evolution of new dependencies that are costly [47, 65]. One high G-score mutation in *Mm* (MMP1511) could reflect the evolution of a new costly dependency. Alanine was earlier shown to be exchanged between the two interacting partners during syntrophic growth, likely at a cost to the producer (*Dv*) and of energetic advantage to *Mm* [66]. Alanine production by *Dv* provides a mechanism to re-oxidize reduced internal cofactors during syntrophic growth, but at the cost of a high-energy phosphate bond. In turn, alanine taken up and converted to pyruvate and ammonia by *Mm* serves as both a carbon and a nitrogen source, alleviating complete dependency on energetically costly autotrophic growth with hydrogen. A cheater population, e.g., one with a loss of function mutation in MMP1511, might consume additional alanine through passive transport and therefore consume less hydrogen to maintain lactate consumption by *Dv*. Indeed, mutations in MMP1511 rose to fixation in six out of nine lines, and we cannot rule out if minor MMP1511 mutant populations also exist in low frequency in the other lines, including UE3.

The observation that interactions among some genotypes were more productive than other pairings suggests that the enhanced cooperativity of evolved communities could have occurred through the selection of complementary mutations across *Dv* and *Mm*, invoking the possibility of partner choice and partner fidelity feedback [67]. Furthermore, each EPD had significantly better growth characteristics than any of the pairings of their member clonal isolates, demonstrating the emergence of increased cooperativity from guilds or “collections of genotypes” of *Dv* and *Mm*. In conclusion, the multiscale dissection of independent laboratory evolution lines has demonstrated that selection of complementary mutations across *Dv* and *Mm* synergistically increased the cooperativity and productivity of syntrophic interactions within both SR− and SR+ communities, while supporting their coexistence in vastly different proportions as *r*- and *K*-strategists, respectively.

## Methods

### Strains and culture conditions

All the strains, culture conditions, and the setup of the laboratory evolution experiment were the same as described before [4, 5]. (See Supplementary Methods for details).

### Sequencing of evolved cocultures

DNA was extracted with Masterpure Kit (Epicentre, WI, USA), processed for sequencing using Nextera DNA library preparation kit (Illumina, CA, USA) and sequenced on Illumina Hiseq with 100 bp paired-end sequencing or on Illumina MiSeq in the paired-end mode producing 2 × 250 bp long reads as described before [5].

### Identification of mutations in evolved cocultures

Mutations in populations were determined using a custom sequence alignment and variant calling pipeline (https://github.com/sturkarslan/evolution-of-syntrophy) (Supplementary Methods). Variant calling was performed with GATK UnifiedGenotyper [34], Varscan [33] (version 2.3.9) and bcftools from Samtools [68] package. Variants identified by each caller were collated and filtered for variant frequency ≥20%. Variants called by at least two algorithms were included for further analysis, including annotation using SnpEff (version 4.3) [69].

### Single-cell sequencing

Single cells of *Dv* and *Mm* from mid-log phase EPD cultures were sorted into wells of a 96-well plate and lysed by using a freeze-and-thaw cycle. Whole Genome Amplification from single cells was performed using REPLI-G Single-Cell kit (Qiagen, MD, USA). We screened single amplified genomes (SAGs) with 16S universal primers for *Dv* and *Mm*, and used AmpPure XP magnetic beads (Beckman-Coulter, CA, USA) to clean and purify confirmed SAGs; QC was performed with Bioanalyzer. DNA concentrations were determined using Quant-iT PicoGreen dsDNA assay kit (Thermofisher, MA, USA). Sequencing was performed as described above.

### Single-cell lineage tree building

Variants detected in at least two cells at >80% frequency were converted into a matrix (1 = mutation present, 0 = mutation absent, or 3 = not enough reads) with unique mutation in rows and single cells in columns. Mutation histories of single cells were determined using the SCITE algorithm [36] with parameters -r 1 -l 90,0000 -fd 6.04e-5 -ad 0.21545 0.21545 -cc 1.299164e-05. Temporal ordering of mutations was cross-referenced with longitudinal sequencing data for the UE3 line, especially when there was ambiguity due to noisy and missing data.

### Calculation of G-scores

G-score (“goodness-of-fit”) for each gene was calculated based on the frequency of observed nonsynonymous mutations (normalized to gene length and genome size) across 13 evolved lines, as described before [18]. G-scores for all genes in the genome of each organism were summed to get the “total observed G-statistic” and compared to the simulated “total expected G-statistic” by calculating a *Z*-score as described in Supplementary Methods and in [18].

### Density dilution assay

Ancestral cocultures and EPDs were grown anaerobically in balch tubes containing coculture medium A (CCMA) [64] with 80%:20% N_2_:CO_2_ headspace at 30 °C without shaking. All stationary phase cultures were diluted to the same OD_600_ value and subjected to a 1.5-fold dilution series in 96-well plates. Blank media were included in the first column of each 96-well plate to rule out contamination. All steps were performed in a Coy Anaerobic Chamber with 95%:5% N_2_:H_2_. Growth measurements were performed in a plate reader (BioTek, VT, USA). See Supplementary Methods for more details.

### Clonal isolate pairings and measurement of growth rate and yield

All *Dv* clonal isolates were revived anaerobically in balch tubes containing CCMA flushed with 80%:20% N_2_:CO_2_. *Mm* isolates were revived in balch tubes pressurized to 30 psig with 80%:20% H_2_:CO_2_. Aliquots (0.1–0.2 ml) of stationary phase cultures (after two transfers) of *Dv* and *Mm* were combined in 20 ml of CCMA containing 30 mM sodium lactate in balch tubes flushed with 80%:20% N_2_:CO_2_, and incubated at 37 °C with shaking at 300 rpm. Growth parameters were estimated by analyzing changes in cell density (OD_600nm_) using grofit [70].

### Excess over Bliss analysis for measuring synergy

Synergistic, additive, and antagonistic effects of clonal isolate pairings was determined using the Bliss Independence model [71]. The experimentally measured fractional growth rate and yield for *Dv* (*f*_*Dv*_) and *Mm* (*f*_*Mm*_) was determined by pairing evolved clonal isolates with ancestral clones. The expected fractional effect on growth rate and yield *f*_*DvMm*_, induced by the combined effect of evolved isolates was calculated as:

$$f_{DvMm} \,=\, 1 \,-\, \left( {1 \,-\, f_{Dv}} \right) \times \left( {1 \,-\, f_{Mm}} \right) \,=\,f_{Dv} \,+\, f_{Mm} \,-\, f_{Dv} \times f_{Mm}$$

Excess over Bliss (EOB) was determined by computing the difference in fractional improvement of growth rate or yield induced by combination, *f*_*z*_, and the expected fractional inhibition, *f*_*DvMm*_.

$$EOB \,=\, \left( {f_z \,-\, f_{DvMm}} \right) \times 100$$

A clonal isolate pair combination for which EOB ≈ 0 has an additive behavior, whereas a pair with positive or negative EOB values has synergistic or antagonistic behavior, respectively. Error was computed by propagating the standard deviation of fractional effects.

### Statistical analysis of mutation emergence across evolutionary trajectories

To quantify the likelihood of observing a mutated gene given another one mutated, we generated a background distribution assuming a mutation in a particular gene as an independent event. We simulated a large random set of mutational trajectory experiments maintaining the number of mutations per evolutionary line and the mutational frequency observed for each gene. We computed an empirical *P* value of an experimental observation as *P* = (*s* + 1)/(*n* + 1), where *s* is the number of the simulation instances with equal or stronger association as observed experimentally and *n* is the number of simulated mutational trajectory experiments (*n* = 1 × 10^6^).

## Supplementary information

Supplemental Material

## Data Availability

All sequencing data are available in NCBI Bioproject database (accession number: PRJNA248017).

## Code availability

Custom R and Python codes are available on GitHub (https://github.com/sturkarslan/evolution-of-syntrophy). Annotated mutations within the context of other functional and regulatory genome information can be explored through Syntrophy Portal (http://networks.systemsbiology.net/syntrophy/).
