# Supplementary material for: Synergistic epistasis enhances the co-operativity of mutualistic interspecies interactions
Source: ISME J. 2021 Feb 21;15(8):2233–47. doi: 10.1038/s41396-021-00919-9 (PMC8319347; doi:10.1038/s41396-021-00919-9)
Supplement: Supplementary file 1 — Supplemental Material [file 41396_2021_919_MOESM1_ESM.pdf]

SUPPLEMENTARY INFORMATION

Supplementary Methods. Supplementary Materials/Subjects and Methods.

Supplementary Figure 1. HR2 lineage tree with mutations mapped to branches. All mutation data (except early generations) for HR2 including 1K generation, EPDs and Clonal isolates were used to discover lineage relationships between EPDs by using siFit algorithm [68] and tree was plotted by using IcyTree [69]. **A)** Detailed tree **B)** Simplified tree with lower tree branches collapsed.

Supplementary Figure 2. Comparison of *Dv* mutations identified in ancestral cocultures and accumulated in UE3 line across, 1K generation, EPDs and Clonal isolates. The length of bar plots denotes frequency for each mutation (rows) while color indicates different mutations.

Supplementary Figure 3. Comparison of *Mm* mutations identified in ancestral cocultures and accumulated in UE3 line across, 1K generation, EPDs and Clonal isolates. The length of bar plots denotes frequency for each mutation (rows) while color indicates different mutations.

Supplementary Figure 4. Density dilution assay growth curves to quantify cooperativity. Using 1.5 serial dilutions in 96-well plates, the Ancestor (Anc), EPD-03, and EPD-09 were grown anaerobically in plate reader and growth was followed by OD measurements as described in the Methods. Dilution factors are indicated as panel titles.

Supplementary Figure 5. Growth rate, yield and cooperativity of EPDs, and clonal isolate pairings.

Supplementary Table S1: Mutations identified in *Desulfovibrio* (*Dv*) and *Methanococcus* (*Mm*) in Early Generations, 1K Generation, EPDs, and Clonal isolates.

Supplementary Table S2: Mutations overlapping across 1K generation, EPDs and Clonal Isolates for evolution lines UE3 and HR2 of *Dv* and *Mm*

Supplementary Table S3: Mutations identified in D*esulfovibrio* (*Dv*) and *Methanococcus* (*Mm*) in single cells of EPD-03 and EPD-09

Supplementary Table S4: Growth rate and yield for clonal isolate pairings

SUPPLEMENTARY METHODS

Strains and Culture Conditions.

All the strains, culture conditions and the setup of the laboratory evolution experiment were the same as described before (Hillesland and Stahl, 2010, Hillesland et al., 2014). Briefly, two clones of *Desulfovibrio (Dv)* and *Methanococcus (Mm)* were paired to setup 24 ancestral cultures in coculture medium A (CCMA) (Stolyar et al., 2007) under anaerobic conditions (80% N_2:20% CO_2 headspace) in Balch tubes. Cocultures were propagated weekly into a fresh media through 100-fold dilutions and incubated either upright without shaking or in a horizontal position with constant shaking at 300 rpm. Laboratory evolution experiment was continued for 152 weeks and populations were archived as frozen glycerol stocks after generations 100, 300, 500, 780, and 1000 generations. Biomass collection was done as described before (Hillesland et al., 2014).

Sequencing of Evolved Cocultures.

DNA sequencing was performed for 13 of 22 evolved cocultures after 1000-generation and 9 evolved cocultures were sequenced at 100, 300, 500 and 780-generations. In addition, three End-Point-Dilutions from UE3 (EPD-03, EPD-09 and EPD-10) and HR2 (EPD-01, EPD-05 and EPD-10) cocultures evolved for 1000 generations and 3 clones of *Dv* and *Mm* from each of these EPDs were sequenced. For each sample, DNA was extracted with Epicentre Masterpure Kit (Epicentre Catalog number: MC85200). Sample and sequencing library preparation was done by using the Nextera DNA library preparation kit (Illumina) according to the manufacturer's instructions. DNA sequencing was performed in an Illumina Hiseq (generations 100, 300, 500, and 780) with 100 bp paired end sequencing or in an Illumina MiSeq sequencing instrument in the paired-end mode producing 2x250 bp long reads as described before (Hillesland et al., 2014).

Identification of Mutations in Evolved Cocultures.

Mutations accumulated in populations were determined by using a custom sequence alignment and variant calling pipeline (https://github.com/sturkarslan/evolution-of-syntrophy). This pipeline included quality control and trimming of the raw sequencing reads in fastq format by using Trim Galore software (http://www.bioinformatics.babraham.ac.uk/projects/trim_galore). The alignment of the quality trimmed sequences to reference *D. vulgaris* (Genbank assembly: GCA_000195755.1.30) and *M. maripaludis* (Genbank assembly: GCA_000011585.1) genomes and subsequent processing steps before calling the variants was done by following The Genome Analysis Toolkit (GATK) (DePristo et al., 2011) best practices. Briefly, reads were first aligned to

the reference genome using Burrows-Wheeler Alignment Tool (bwa) (Li and Durbin, 2009) (version 0.7.17-r1188) in paired-end mode. The resulting alignment files in the SAM format were converted to BAM files, sorted and indexed by using Samtools version 1.9 (Li et al., 2009). BAM files were marked for duplicates using Picard Tools (http://broadinstitute.github.io/picard/) (version 1.139), and local realignment around indels was performed to identify the most consistent placement of reads relative to the indels. Variant calling was performed independently by using three different algorithms including GATK UnifiedGenotyper, Varscan (Koboldt et al., 2012) (version 2.3.9) and bcftools from Samtools package. The default parameters were used for UnifiedGenotyper, whereas for Varscan parameters were --min-coverage 8 --min-reads2 2 --min-avg-qual 30 and bcftools parameters were -vmO -s LOWQUAL -i'%QUAL>30. Variants identified by each caller were collated and filtered for variant frequency equal or greater than 20%. A variant was included in the analysis only if it is simultaneously called by at least two of the callers. The resulting variants were annotated using SnpEff tools (Cingolani et al., 2012) (version 4.3).

Single Cell Sequencing

For single cell sequencing, EPD-03 and EPD-09 from UE3 evolved cocultures were grown to mid-log phase. Single cells of *Desulfovibrio* or *Methanococcus* were sorted into wells of a 96-well plate containing 3 µl of PBS and Buffer D2 from Repli-G single cell kit (Qiagen) by using Influx flow cytometer (BD). For each EPD, one plate for each of *Desulfovibrio* and *Methanococcus* was prepared. In order to lyse the cells, a freeze-and-thaw cycle was performed by first spinning the plates and freezing them at -20°C followed by thawing and re-spinning. Whole Genome Amplification (WGA) from single cells was performed by using REPLI-G Single Cell kit (Qiagen) according to manufacturer's instructions. We screened single amplified genomes (SAGs) with 16S universal primers for *Desulfovibrio* or *Methanococcus* to identify percentage of wells that did not contain any amplified product due to missing cells or failed WGA reaction. Wells with confirmed amplification were further treated with AmpPure XP magnetic beads (Beckman-Coulter) to clean and purify SAGs. A subset of SAGs was also analyzed with Bioanalyzer to confirm the size of the amplified fragments. Concentration of the SAGs passing the quality controls were determined by using Quant-iT PicoGreen dsDNA assay kit (Thermofisher). Nextera XT Library preparation kit (Illumina) was used for sample and library preparation for sequencing. Sequencing was performed in HiSeq platform (Illumina) by using High-Output flow cell in 2x150 bp paired-end format. Sequence analysis including quality controls, trimming, alignment and variant calling was performed as described above.

Single Cell Lineage Tree Building

Variants identified from single cells of *Desulfovibrio* or *Methanococcus* for EPD-03 and EPD-09 of 1000-generation evolved UE3 cocultures were converted into a binary mutation matrix where each row was a unique mutation and each column was a single cell. Values in the binary mutation matrix were either 1 (mutation was seen in that particular cell), 0 (mutation was not observed) or 3 (there wasn't enough confident reads to assign the mutation). A variant was considered for the analysis only if its frequency was over 80% and was seen in at least two single cells. Mutation histories of single cells were determined by using SCITE algorithm (Jahn et al., 2016) with parameters -r 1 -l 900000 -fd 6.04e-5 -ad 0.21545 0.21545 -cc 1.299164e-05. SCITE used stochastic search to find the Maximum Likelihood tree of mutation histories in Newick format, which was converted to Cytoscape format for visualization purposes. This tree represents the predicted temporal order of the mutation events. Mutations were re-ordered by using information from the sequencing of the early generation cocultures if the order of the mutations couldn't be determined from the single cell mutational profiles due to noisy and missing data. Mutation tree was further annotated with gene functions, type of mutations and status of the mutations in early generations, and clonal isolates.

Calculation of G-scores

Based on the frequency of observed mutations (normalized to gene length and genome size) across 13 evolved lines, we calculated a G-score ("goodness-of-fit") to assess if the observed parallel evolution rate was higher than background as described before (Tenaillon et al., 2016). Briefly, expected number of mutations (E_i) for each gene in the genome was calculated as:

$$E_i = N_{tot}(L_i/L_{tot})$$

where N_{tot} is the total number of mutations, L_i is the length of the gene i and L_{tot} is the total length of the coding genome. G-score for each gene (G_i) was calculated as:

$$G_i = 2N_i \log_e(\frac{N_i}{E_i})$$

where N_i is the number of nonsynonymous mutations observed for gene i across all evolved lines. G-scores for all genes in the genome of each organism were summed up to get the "total observed G-statistic" (G_{obs}). In order to get the "total expected G-statistic" (G_{exp}), we simulated N_{tot} number of mutations randomly across the protein-coding genome and calculated the mean and standard deviation of G-statistic from all simulations. We compared the observed and expected G-statistics by calculating a Z-score as follows;

$$Z = (G_{obs} - \mu(G_{exp}))/\sigma(G_{exp})$$

Where $\mu(G_{exp})$ and $\sigma(G_{exp})$ are the mean and standard deviation of the G-statistics from 1000 simulations, respectively.

Density dilution assay

Ancestor and EPD cocultures were revived anaerobically in 18×150-mm balch tubes (Chemglass Life Sciences: CLS420901) from freezer stocks through dilution into CCMA media to ensure syntrophic growth and prevent carryover of glycerol into the fresh growth medium. EPD batch cultures (10 mL) were grown in anaerobic conditions with 80%:20% N_2:CO_2 headspace at 30°C without shaking. Growth of the cultures were monitored using a spectrophotometer (Spectronic 200: Fisher Scientific) to measure the optical density at 600 nm (OD600), measurements were typically taken twice a day until the cocultures reached stationary phase (~0.7 for EPD-03 and EPD-09). The cultures were kept in stationary-phase for approximately 20 hours in order to ensure similar growth phases before dilution into 96-well plates (Thomas Scientific: 1154Q44) for the density dilution assay. Stationary-phase Ancestor and EPD cultures were all diluted to the same starting optical density (as measured by the 96-well plate reader: BioTek EPOCH2T), so that all cultures in column 12 of each plate have the same starting optical density. Thereafter, each column between 11 and 2 received a volume of cells that would equate to a 1.5-fold dilution of the previous column and starting with column 11. The 1st column of each 96-well plate contained only media as a control to identify potential contamination. All dilutions of cocultures into plates for the density dilution assay were done inside a Coy Anaerobic Chamber with an approximate atmospheric ratio of 95%:5% N_2:H_2. Plates were sealed with optically clear strong adhesive PCR films (115x100mm, Thomas Scientific: 4ti-0500/8), and the edges of these seals were coated twice in clear acrylic to further inhibit potential gas diffusion into the wells. Following inoculation, sealed plates were incubated at 30°C within the anaerobic chamber. Plates were removed from the anaerobic chamber twice per day to take growth measurements in the BioTek plate reader. Plates in the plate reader were shaken linearly for 5 seconds prior to OD600 measurements at 30°C. During the transfer of plates from anaerobic chamber to plate reader, they were insulated between two 6-well plates filled with H_2O that were also incubated at 30°C in order to maintain constant temperatures during plate transport from anaerobic chamber to plate reader. Density dilution assays were carried out for approximately ~4-5 days. A moving average with a window of two was applied to ODs for each density dilution assay to smooth the timeseries data. In order to establish a baseline for growth, a threshold was calculated for each EPD and ancestor strain based on the minimum carrying capacity from the first two dilutions (n=16). If cells from a well did not achieve an OD of that minimal threshold or higher, those cells were considered as not grown.

Clonal isolate pairings and measurement of Growth Rate and Yield.

Isolates of *Desulfovibrio* from EPD-03 or 09 of line UE3 were revived anaerobically using 10 ml of CCMA containing 10 mM:7.5 mM sodium lactate:sodium sulfite or 30 mM:20 mM sodium lactate:sodium sulfate, respectively, in balch tubes flushed with 80%:20% N_2:CO_2. Isolates of *Methanococcus* were revived anaerobically using 5 ml of CCMA containing 10 mM sodium acetate in balch tubes pressurized to 30 psig with 80%:20% H_2:CO_2. After the second transfer of revived isolates on their respective media, 0.1-0.2 ml of stationary phase cultures were combined in 20 ml of CCMA containing 30 mM sodium lactate in balch tubes flushed with 80%:20% N_2:CO_2. After the second transfer, cocultures were stored as freezer stocks for future growth analysis. Cocultures for growth analysis were revived anaerobically using 20 ml of CCMA containing 30 mM sodium lactate in balch tubes flushed with 80%:20% N_2:CO_2. Cultures were incubated at 37°C and shaken horizontally at 300 rpm. Optical densities (OD_{600nm}) were monitored to assess growth and growth parameters were estimated using the fitting package grofit (Kahm et al., 2010).

Excess over Bliss analysis for measuring synergy

We adapted the Bliss Independence model (Borisy et al., 2003) to predict if accumulated mutations in evolved *Dv* and *Mm* partners have an additive effect on growth rate and yield of their clonal isolate pairings. The experimentally measured fractional growth rate and yield for *Dv* (f_{Dv}) and *Mm* (f_{Mm}) was determined by pairing their evolved clonal isolates with ancestral clones of their respective partners. Then, the expected fractional effect on growth rate and yield f_{DvMm}, induced by the combined effect of evolved isolates was calculated as:

$$f_{DvMm} = 1 - (1 - f_{Dv}) \times (1 - f_{Mm}) = f_{Dv} + f_{Mm} - f_{Dv} \times f_{Mm}$$

Excess over Bliss (EOB) was determined by computing the difference in fractional improvement of growth rate or yield induced by combination, f_z, and the expected fractional inhibition, f_{DvMm}

$$EOB = (f_z - f_{DvMm}) \times 100$$

A clonal isolate pair combination for which EOB ≈ 0 has an additive behavior, whereas a pair with positive or negative EOB values has synergistic or antagonistic behavior, respectively. Error bars were computed by propagating the standard deviation of fractional effects.

Data and Code availability

Bulk and Single cell sequencing data used in this study and associated biosample meta-data information can be obtained through the NCBI Bioproject database (https://www.ncbi.nlm.nih.gov/bioproject) with accession number PRJNA248017.

Custom R and Python codes used for sequence analysis, variant calling, data analysis and figure preparations are available on GitHub (https://github.com/sturkarslan/evolution-of-syntrophy).

Annotated mutations within the context of other functional and regulatory genome information can be explored through Syntrophy Portal (http://networks.systemsbiology.net/syntrophy/)

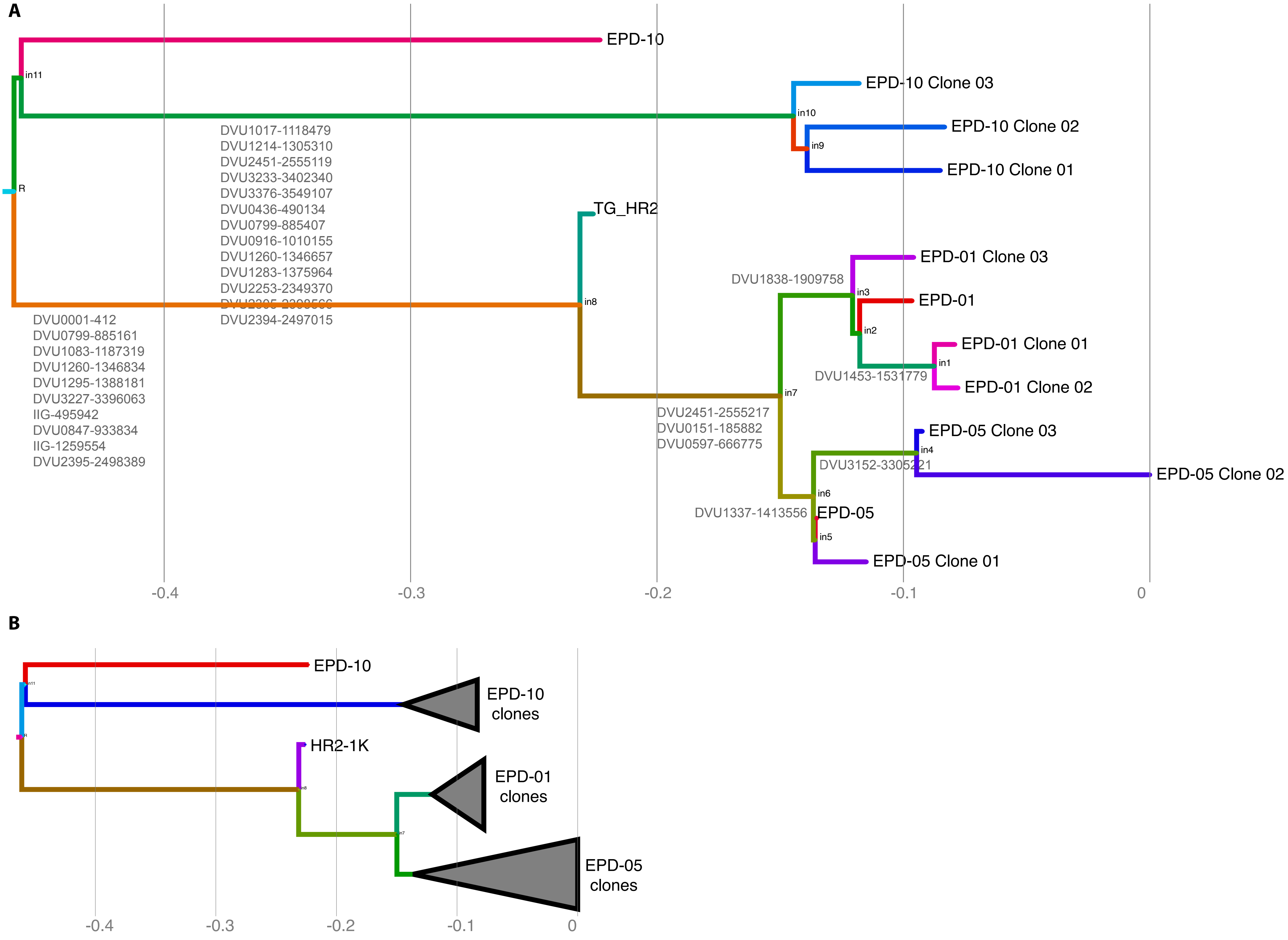

Supplementary Figure 1

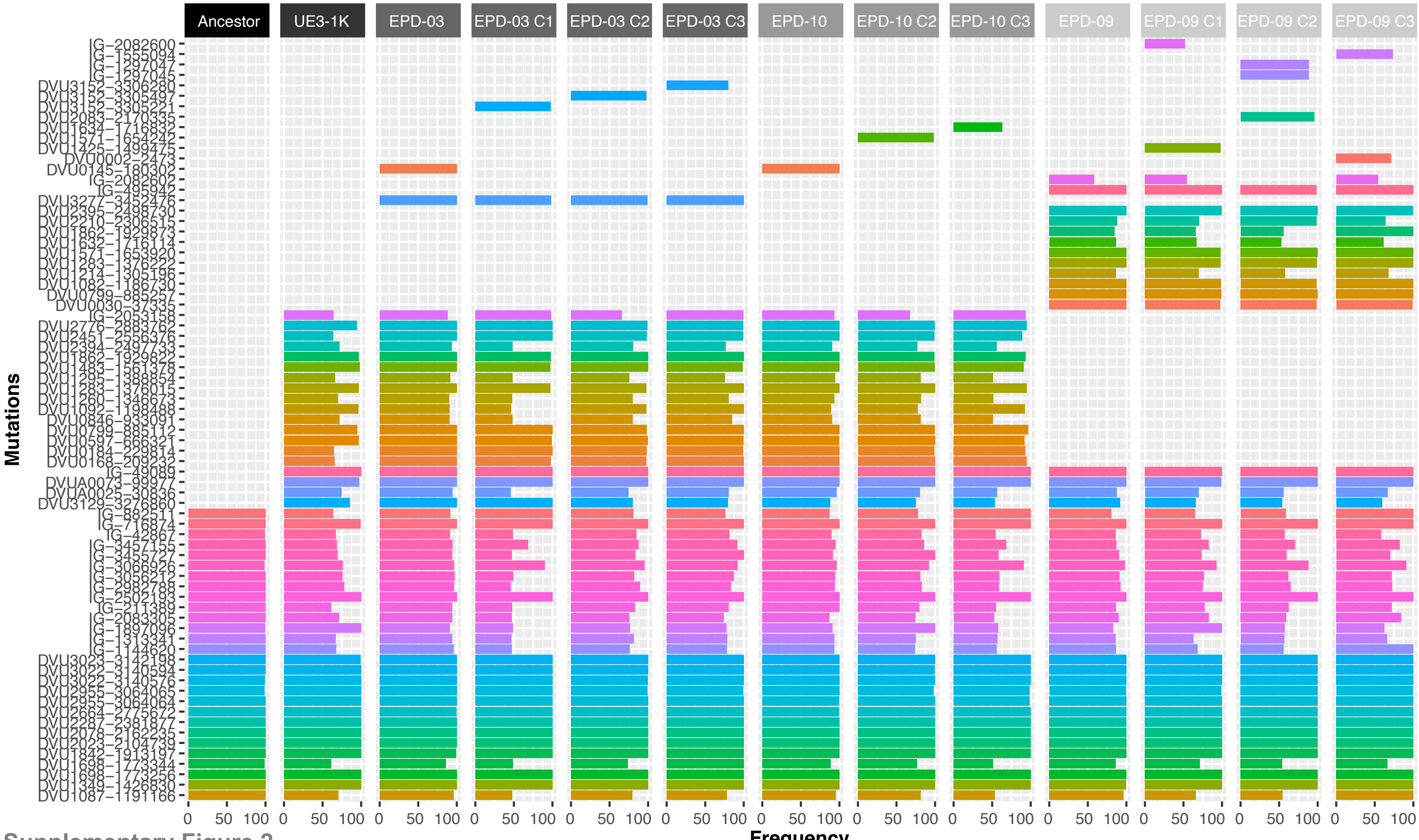

Supplementary Figure 2

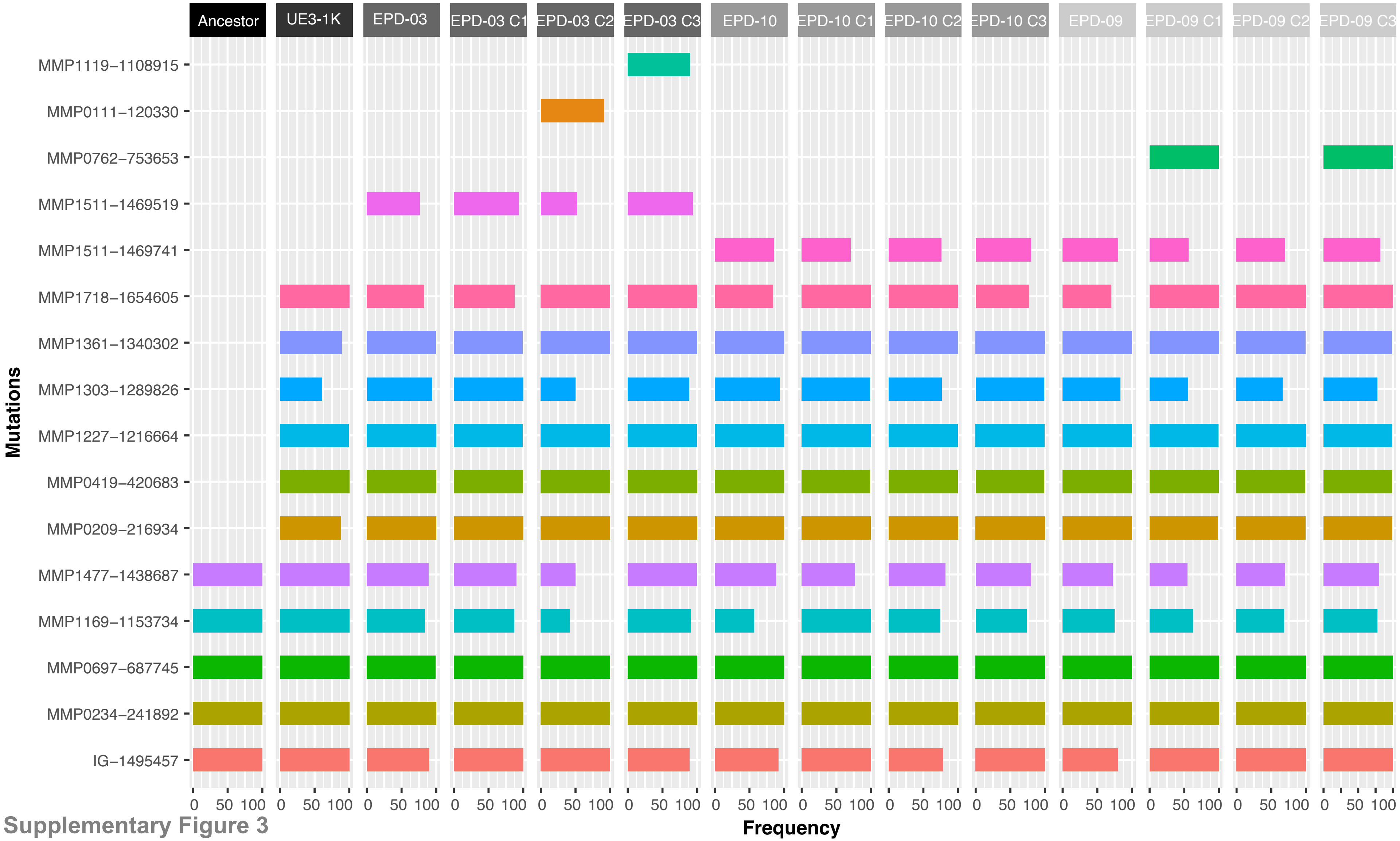

Supplementary Figure 3

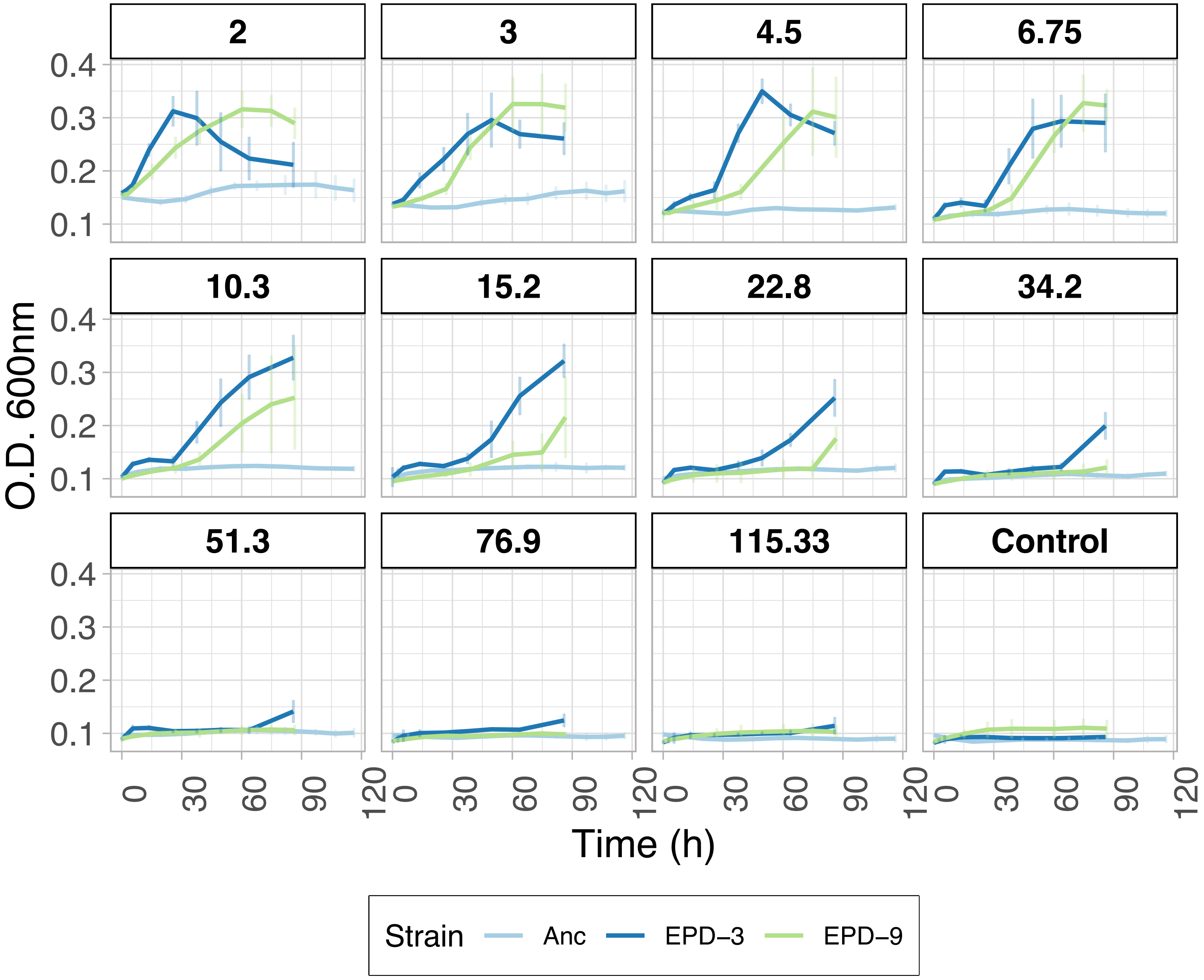

Supplementary Figure 4

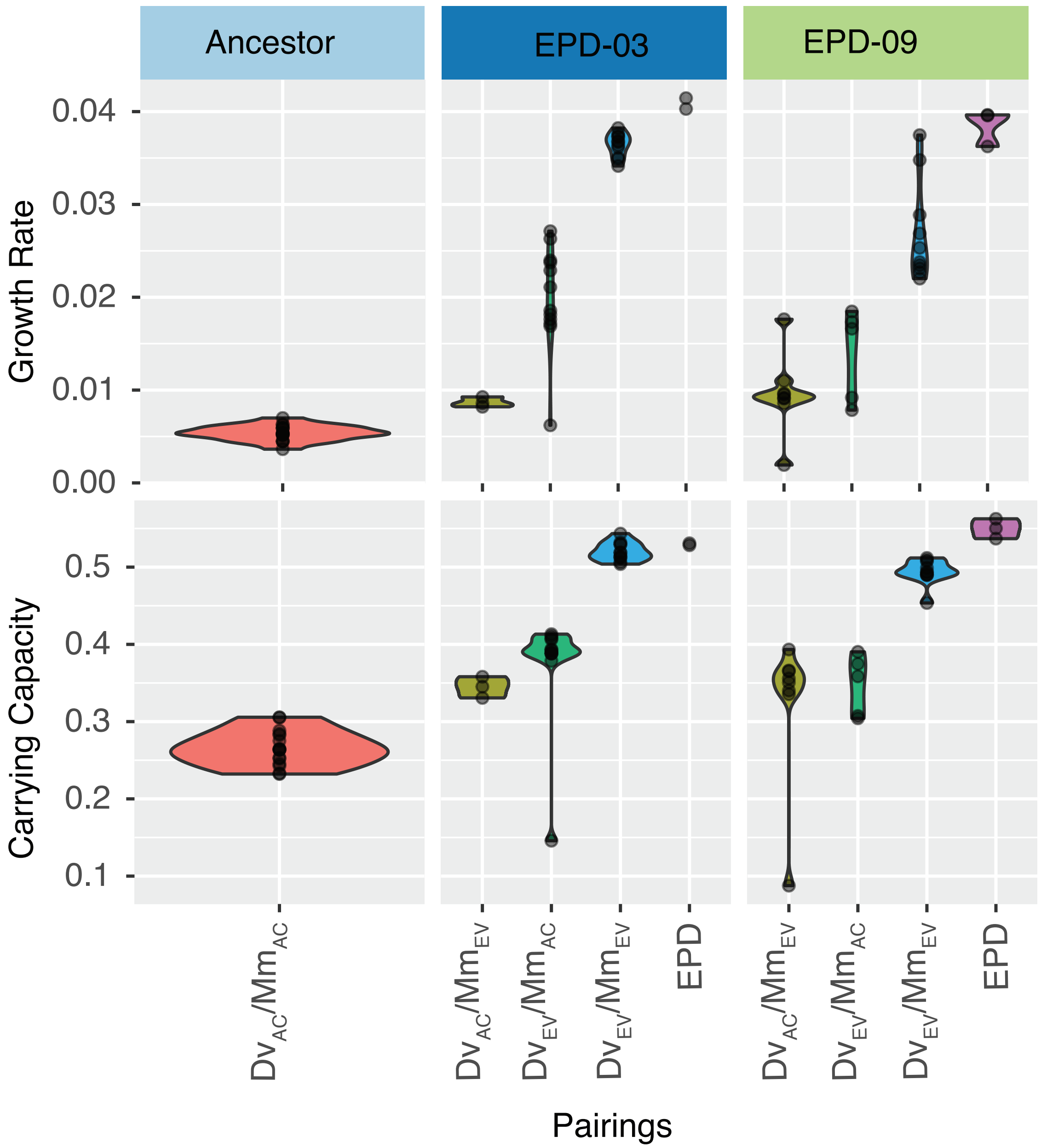

Supplementary Figure 5
